# An optical coherence tomography study of a photoactive Pt(iv) prodrug in oesophageal tissue†

**DOI:** 10.1039/d4ra08693g

**Published:** 2025-01-31

**Authors:** Huayun Shi, Muktesh Mohan, Kanwarpal Singh, Peter J. Sadler

**Affiliations:** a Department of Chemistry, University of Warwick Coventry CV4 7AL UK Huayun.Shi@xmu.edu.cn p.j.sadler@warwick.ac.uk; b State Key Laboratory of Vaccines for Infectious Diseases, Center for Molecular Imaging and Translational Medicine, Xiang An Biomedicine Laboratory, School of Public Health, Xiamen University Xiamen 361102 China; c State Key Laboratory of Molecular Vaccinology and Molecular Diagnostics, National Innovation Platform for Industry-Education Integration in Vaccine Research, Xiamen University Xiamen 361102 China; d Max-Planck-Institut für die Physik des Lichts Staudtstraße 2 91058 Erlangen Germany kanwarpal.singh@mpl.mpg.de; e Max-Planck-Zentrum für Physik und Medizin Kussmaulallee 2 91054 Erlangen Germany; f Department of Electrical and Computer Engineering, McMaster University 1280 Main Street West Hamilton ON L8S 4K1 Canada

## Abstract

Photoactive diazido Pt(iv) complexes display *in vivo* anticancer efficacy towards oesophageal tumours, a worldwide common cancer. Here we explore the use of optical coherence tomography (OCT) as a new method for detecting tissue penetration and damage produced by the photoactivatable anticancer complex *trans*,*trans*,*trans*-[Pt(pyridine)_2_(N_3_)_2_(OH)_2_] (FM190). Dehydration of the sample and a change in refractive index were observed for swine oesophageal tissue treated with FM190 and blue laser light (445 nm) using an OCT system. In contrast, tissues treated with FM190 or laser light alone showed no apparent damage.

## Abstract 2

Reflected laser-light images show that this photoactive Pt(iv) complex penetrates oesophageal tissue and causes morphological damage only when irradiated.

## Graphical abstract

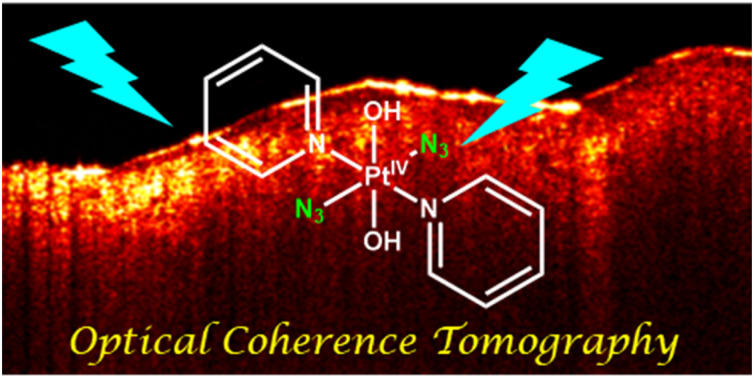

Oesophageal cancer is the ninth most common cancer and the sixth leading cause of cancer deaths worldwide.^1^ Phototherapy is a highly controllable treatment for cancer spatially and temporally with minimal invasiveness.^2–6^ Photofrin-based photodynamic therapy (PDT) was approved in Japan to treat oesophageal cancers in 1994.^7,8^ Compared with surgery, PDT is less invasive and results in a better quality of life.^7^ However, oxygen-dependent mechanism limits the application of PDT in hypoxic tumours.^9^ Photoactive diazido Pt(iv) complexes are emerging anticancer prodrugs with advantages, including high dark stability and promising photocytotoxicity with visible light under both normoxia and hypoxia.^10,11^ Two of seven nude mice bearing an OE19 oesophageal cancer xenograft treated with *trans*,*trans*,*trans*-[Pt(pyridine)(NH_3_)(N_3_)_2_(OH)_2_] at low dose with short irradiation times (420 nm) survived after 35 days, while none of the control mice treated without drug or irradiation survived.^12^*Trans*,*trans*,*trans*-[Pt(pyridine)_2_(N_3_)_2_(OH)_2_] (FM190, Fig. 1a) is the current lead compound of photoactive diazido Pt(iv) prodrugs, which can undergo photosubstitution and photoreduction to release azidyl radicals and activated Pt(ii) and Pt(iv) species to kill cancer cells with blue light.^13–15^

**Fig. 1:**
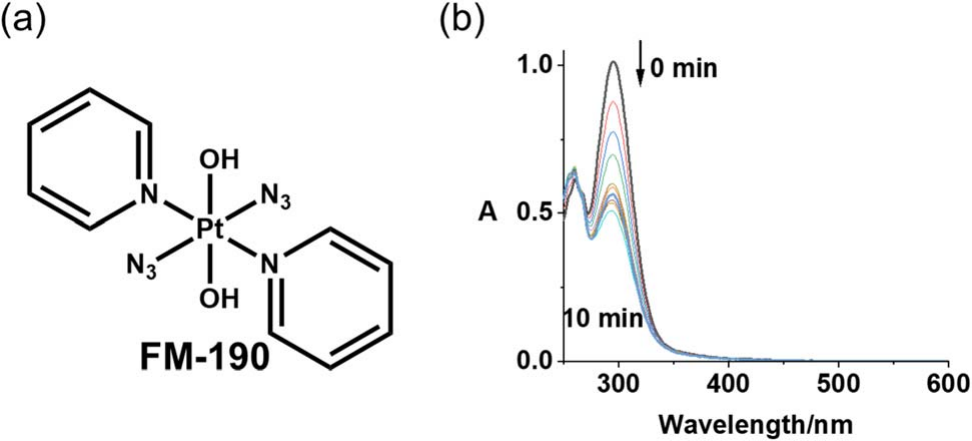
(a) Chemical structure of FM190; (b) UV-vis spectral changes for FM190 in water exposed to blue laser light (445 nm) at 298 K, time intervals are 1 min. The decrease in intensity of the ligand (azide)-to-metal (Pt(iv)) charge transfer band at 298 nm is indicative of the breaking of Pt–N_3_ bonds and reduction of the relatively inert octahedral Pt(iv) complex to more reactive square-planar Pt(ii) species.

Complexes in this class of Pt(iv) prodrugs are best activated by blue or green light which travels only *ca.* 1–3 mm into tissue.^16^ This makes them well suited for use in therapy of surface cancers such as bladder and oesophageal. Although red light with longer wavelength can penetrate more deeply (*ca.* 5 mm), this increases the risk of damage to deeper underlying normal tissue when treating surface cancers.^17^

We are therefore assessing methods, which can investigate the effects of these complexes on surface tissues both in the dark and in the light. The imaging technique of optical coherence tomography (OCT) uses short-coherence-length light and interferometry together with transverse scanning of the light beam to produce two- and three-dimensional images from light reflected from within biological tissues. Since its first use in 1991, it has become widely used for medical diagnosis, especially in ophthalmology for monitoring retinal, optic nerve and corneal diseases.^18^ It can rapidly achieve subsurface imaging at depths of 1–2 mm with simple sample preparation, and high-resolution subcellular micrometre resolution at low-cost.^19–21^

Lasers with high power output and easy connection to optical fibres and endoscopes can provide precise light delivery to tumours.^17^ The FM190 used in this work was synthesized and characterized as previously reported,^13^ and had a purity of >99%. The photoactivation of FM190 in aqueous solution with 445 nm laser light was very rapid due to the high intensity of light (250 mW cm^−2^), and was complete within 10 min (Fig. 1b).

The effect of FM190 on swine oesophageal tissue in the presence and absence of laser light exposure was determined using OCT. A phosphate-buffered saline (PBS) solution (200 μL) of FM190 with different concentrations (0, 100, 500 and 1000 μM) was dropped onto the mucosa of a piece of swine oesophageal tissue (*ca*. 1 × 1 cm^2^), which was then incubated at 310 K in the dark for 2 h. The tissue was scanned by the OCT system to obtain the morphology in the dark. After 10 min irradiation with blue laser light (445 nm), the same area of this tissue was scanned again to determine the morphology after light exposure.

The attenuation coefficient is a crucial parameter in OCT signal analysis. It is a measure of the decay of light intensity within the sample due to absorption and scattering, and can be calculated with the aid of the Beer–Lambert law, which can be expressed as eqn (1).^22,23^

1 *I* = *I*_0_ e^−2(*μ*_s_ + *μ*_a_)*z*^

where *I* is the intensity of light represented by the A-scan profile, *I*_0_ is the peak intensity at the surface of the sample, *μ*_s_ is scattering coefficient, *μ*_a_ is the absorption coefficient, and *z* is the imaging depth multiplied by 2 for the round-trip journey of the imaging light beam. Attenuation coefficient *μ*_A_ is the sum of the scattering and absorption coefficients (eqn (2)).

2 *μ*_A_ = *μ*_s_ + *μ*_a_

To evaluate the attenuation coefficient, we extracted the optical characteristics of the incident light beam at any spot over the tissue surface, which involved extracting the optical characteristics of the incident light beam.

During OCT data acquisition, a coherence beam of light is scanned over the sample surface to extract the depth information. Backscattered light preserves the tissue characteristics within it. OCT volumetry data are a collection of A-scans, also known as depth-resolved interferometry signals arranged over the scan surface (Fig. 2). A schematic diagram of the OCT system used in shown in Fig. S1 (ESI†). To evaluate the attenuation coefficient, we extracted an average of 10 A-scans from the B-scan (cross-sectional OCT image) to reduce noise. Part of the A-scan signal consists of tissue characteristic information, which was then exponentially fitted. The coefficient values are equated to the corresponding depth information (see eqn (1)). The attenuation coefficient value was calculated over the whole scanning length of the tissue, and statistical analysis was performed for every sample.

**Fig. 2:**
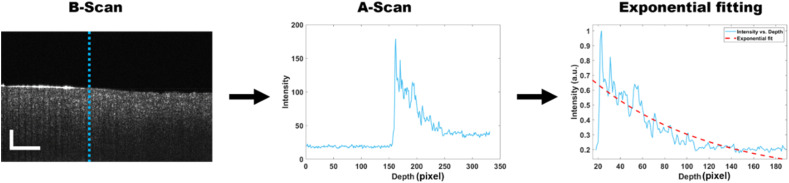
Steps involved in attenuation coefficient calculation. Coefficients for curve-fitted values were divided by the round-trip distance covered by imaging light. Scale bars in B-scan: 500 μm.

After 2 h incubation of swine oesophageal tissue with FM190 in the dark at 310 K, all tissues remained intact and smooth. FM190 acts as a high scattering media for imaging beams of light and thus enhances the contrast of the internal structure (Fig. 3). The enhanced contrast shows that it was absorbed very well deeply into the tissue. When FM190 was activated with a pump laser and interacts with the tissue, this resulted in dehydration of the sample and a change in refractive index. The combined effect changes the morphological structure, as can be observed from comparative images. From the internal microstructures, the field of view becomes restricted due to an increased refractive index, which results in higher attenuation coefficient values. In contrast, the negative control treated with light alone exhibits no apparent changes in morphology and a similar attenuation coefficient compared with the dark control. These results confirm the low dark cytotoxicity of FM190 and damage to the mucosa of swine oesophagus induced by the combination of FM190 and blue laser light. The rapid observation of damage is consistent with the release and formation of reactive azidyl radicals, oxygen species (ROS), as well as photosubtituted Pt(iv) and reduced Pt(iii) and Pt(ii) species.^13,14^ These findings confirm the potential application of FM190 in the treatment of oesophageal cancers as a photoactive prodrug with a new mechanism of action and low side effects.

**Fig. 3:**
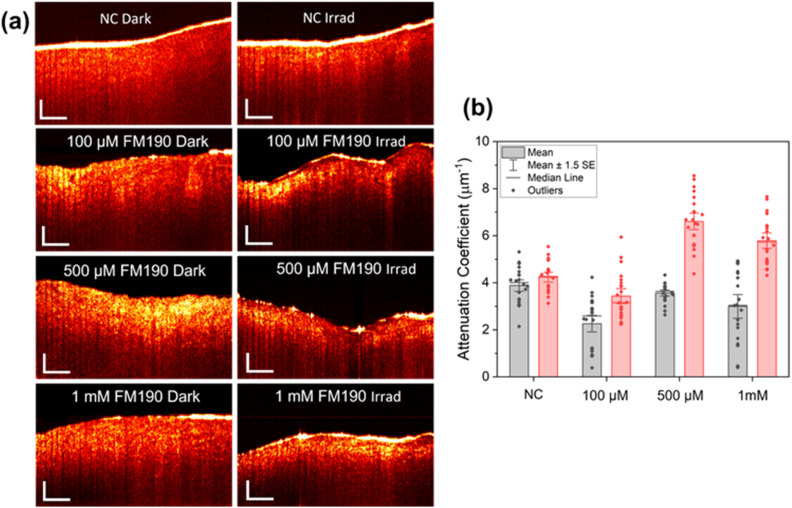
(a) Morphology, and (b) attenuation coefficient values of swine oesophageal tissue treated with FM190 in the dark (2 h) and then irradiated with blue laser light (445 nm, 10 min). NC = negative control. Scale bars in (a): 500 μm.

This is the first exploration of the effect of the photoactivatable Pt(iv) prodrug FM190 on oesophageal tissue in the absence and presence of light using an OCT system. The significant changes in the mucosa of swine oesophagus before and after irradiation suggest the low dark cytotoxicity and promising photocytotoxicity of FM190, which is a promising property of this candidate photoactive anticancer prodrug. The application of OCT technology in the visualisation of the internal structure of biological tissues allows the exploration of drug and light penetration in phototherapy, which can guide the design of new photoactive prodrugs.

## Data availability

The data supporting this article have been included as part of the ESI.†

## Author contributions

H. S., P. J. S. and K. S. designed the experiments. H. S. synthesised and characterised the Pt complex. K. S. and H. S. carried out the imaging and photoirradiation experiments. K. S. and M. M. processed and analysed the imaging data. H. S. drafted the paper and all authors contributed to the final version.

## Conflicts of interest

There are no conflicts of interest to declare.

## Supplementary Material

RA-015-D4RA08693G-s001
