# Supplementary material for: An optical coherence tomography study of a photoactive Pt(iv) prodrug in oesophageal tissue
Source: RSC Adv. 2025 Jan 31;15(5):3168–71. doi: 10.1039/d4ra08693g (PMC11783044; doi:10.1039/d4ra08693g)
Supplement: RA-015-D4RA08693G-s001 [file RA-015-D4RA08693G-s001.pdf]

Supplementary Information (SI) for RSC Advances.
This journal is © The Royal Society of Chemistry 2025

Electronic Supplementary Information

An optical coherence tomography study of a photoactive Pt(IV) prodrug in oesophageal tissue

Huayun Shi[a,b,c*], Muktesh Mohan[d,e], Kanwarpal Singh[d,e,f*], Peter J Sadler[a,*]

[a] Department of Chemistry, University of Warwick, Coventry, CV4 7AL, UK
[b] State Key Laboratory of Vaccines for Infectious Diseases, Center for Molecular Imaging and Translational Medicine, Xiang An Biomedicine Laboratory, School of Public Health, Xiamen University, Xiamen, 361102, China
[c] State Key Laboratory of Molecular Vaccinology and Molecular Diagnostics, National Innovation Platform for Industry-Education Integration in Vaccine Research, Xiamen University, Xiamen, 361102, China
[d] Max-Planck-Institut für die Physik des Lichts, Staudtstraße 2, 91058 Erlangen, Germany
[e] Max-Planck-Zentrum für Physik und Medizin, Kussmaulallee 2, 91054 Erlangen, Germany
[f] Department of Electrical and Computer Engineering, McMaster University, 1280 Main Street West, Hamilton, ON, L8S 4K1, Canada

Experimental section.

Materials and instruments.

Materials.

FM-190 was prepared using the modified synthetic route reported previously.[S1] **FM190** was fully characterized, including UV-vis, ^{1}H, ^{13}C and ^{195}Pt NMR spectroscopy, and ESI-MS.[S1] The ^{1}H NMR and UV-vis spectrum of **FM190** used in the work matched well with our previous report, and the HPLC purity was > 99%. The complex is not luminescent.

Dulbecco's Phosphate-Buffered Saline (PBS) was from Biowest.

Swine oesophagus tissue was from a local (Erlangen) meat market.

Instruments.

Electronic absorption spectra were recorded on a Hitachi U-3900H UV-vis spectrophotometer in a quartz cuvette and solvent used as reference. The spectral width was 200–600 nm and the resolution was 1.0 nm, the scan rate was set to 600 nm/min.

The laser used for irradiation was from Roithner Lasertechnik GmbH, Austria (445 nm, 250 mW/cm^2) and the optical fibre was a multimode fibre (M96L01, 105 micron diameter) from Thorlabs, USA.

Optical coherence tomography system

The current version of the optical coherence tomography system is also known as spectral domain optical coherence tomography (SD-OCT, Figure **S1**). A supercontinuum laser (SC-laser, YSL Photonics, Wuhan, China) is used as a broadband light source, filtered using a short-pass dichroic mirror (DMSP950T, Thorlabs, Newton, NJ, USA), having a central wavelength 850 nm, and was coupled to a single-mode fiber. The laser source operated at 200 MHz,

providing a total width half maximum bandwidth of 80 nm. A 75:25 wideband fiber coupler (TW850R3A2, Thorlabs, Newton, NJ, USA) splits incoming light into a reference path and a sample path. 25% of the input light was used to illuminate the sample, and 75% of light illuminated the reference mirror.

The reference path has a collimator, lens (AC254-030-AB, Thorlabs, Newton, NJ, USA), and a mirror. The sample arm has a collimator, a two-axis galvo scanner (GVS012, Thorlabs, Newton, NJ, USA) controlled via a data acquisition card (DAQ, USB-6211, NI, Austin, TX, USA) for performing raster scanning over the sample surface, a focusing lens and sample stage. Backscattered light from the sample and reflected light from the reference mirror were combined by a fiber coupler and projected on a holographic grating (1200 lines per millimeter, 840 nm, Wasatch, Logan, UT, USA). A lens (AC-254-080-B, Thorlabs, Newton, NJ, USA) was used to focus the light spectra on a line scan camera (2048 pixels, ral2048-48gm, Basler, Ahrensburg, Germany). The spectroscopic signal was acquired using a computer connected via GigE-vision at 25kHz. Further, acquired data sets were processed through a custom-designed LabVIEW program that includes static noise removal, dispersion compensation, spectral reshaping from wavelength space to wavenumber space, and a fast Fourier transform to obtain an axial profile of the sample.

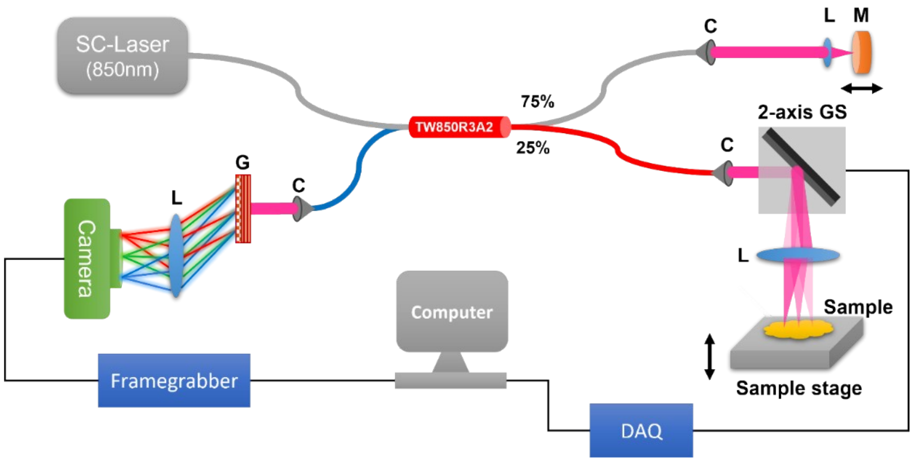

Figure S1. Schematic of Spectral Domain Optical Coherence Tomography (SD-OCT) system. SC-laser: supercontinuum laser, C: collimator, L: lens, M: mirror, 2-axis GS: two-axis galvo scanner, G: holographic grating, DAQ: data acquisition card.[S2]

Photoactivation in solution. The photoactivation of **FM-190** (OD_{300} = 1) in water was monitored by UV-vis spectroscopy at 1 min time intervals upon irradiation with blue laser light (445 nm) at 298 K.

OCT sample preparation. A 200 μL PBS solution of **FM190** with different concentrations (0, 100, 500 and 1000 μM) was dropped into the mucosa of a piece of swine oesophagus tissue (ca. 1×1 cm^2), which was then incubated in the dark at 310 K for 2 h. After obtaining the dark image, the tissue was irradiated with blue laser light (445 nm) for 10 min, and then the same area was scanned for the irradiated image.

References

S1. N. J. Farrer, J. A. Woods, L. Salassa, Y. Zhao, K. S. Robinson, G. Clarkson, F. S. MacKay and P. J. Sadler, *Angew. Chem. Int. Ed.*, 2010, **49**, 8905–8908.

S2. G. Sharma, A. Parmar, F. Hoffmann, K. Geißler, F. von Eggeling, O. Guntinas-Lichius, and K. Singh, *Photonics* , 2024, **9**, 259.
